# Genetic correlation and Mendelian randomization analyses support causal relationships between dietary habits and age at menarche

**DOI:** 10.1038/s41598-024-58999-4

**Published:** 2024-04-10

**Authors:** Ruilong Guo, Ruoyang Feng, Jiong Yang, Yanfeng Xiao, Chunyan Yin

**Affiliations:** 1 Department of Pediatrics, Xi’an Jiaotong University Second Affiliated Hospital, Xi’an, 710054 Shanxi China; 2 Department of Joint Surgery, Xi’an Jiaotong University Hong Hui Hospital, Xi’an, 710054 Shanxi China

**Keywords:** Dietary habits, Age at menarche, Linkage disequilibrium score regression, Mendelian randomization, GWAS, Computational biology and bioinformatics, Genome informatics

## Abstract

Dietary habits are essential in the mean age at menarche (AAM). However, the causal relationship between these factors remains unclear. Therefore, this study aimed to elucidate the genetic relationship between dietary habits and AAM. Genetic summary statistics for dietary habits were obtained from the UK Biobank. GWAS summary data for AAM was obtained from the ReproGen Consortium. Linkage disequilibrium score regression was used to test genetic correlations between dietary habits and AAM. The Mendelian randomization (MR) analyses used the inverse-variance weighted method. Genetic correlations with AAM were identified for 29 candi-date dietary habits, such as milk type (skimmed, semi-skimmed, full cream; coefficient = 0.2704, *P*_*ldsc*_ = 1.13 × 10^−14^). MR evaluations revealed that 19 dietary habits were associated with AAM, including bread type (white vs. any other; OR 1.71, 95% CI 1.28–2.29, *P*_*mr*_ = 3.20 × 10^−4^), tablespoons of cooked vegetables (OR 0.437, 95% CI 0.29–0.67; *P*_*mr*_ = 1.30 × 10^−4^), and cups of coffee per day (OR 0.72, 95% CI 0.57–0.92, *P*_*mr*_ = 8.31 × 10^−3^). These results were observed to be stable under the sensitivity analysis. Our study provides potential insights into the genetic mechanisms underlying AAM and evidence that dietary habits are associated with AAM.

## Introduction

Menarche is a significant sign of pubertal onset, marking the beginning of a female’s fertility and reproductive ability^1^. The age at menarche (AAM) is a well-remembered and widely measured marker of female sexual development. AAM has been widely used in studies of female health^2^, where it has been observed to correlate with a body-mass index (BMI)^3^, height^4^, fertility^5^, psychological health^6^, and cancer^7^. A combination of genetic and environmental factors determines AAM. Large-scale genomic analysis of AAM has identified hundreds of associated variants and determined the genetic mechanism underlying the role of AAM in breast and endometrial cancer risk^8^. However, the effects of environmental factors on AAM remain unclear.

Dietary habits are critical for human health and disease prevention^9^. Unhealthy dietary habits may lead to various diseases, including cardiovascular^10^, endocrine^11^, and infertility^12^. Dietary habits are an important non-genetic factor affecting AAM^13^, and different dietary habits have different effects^14^. Research on the relationship between dietary habits and AAM has focused on observational studies and studies of single food groups. However, a systematic exploration of the potential correlations between dietary habits and AAM remains lacking. It is important to note that most eating habits are correlated and heritable. Genome-wide association studies (GWAS) have been conducted to analyze individual macronutrients in five questionnaires on macronutrient intake^15^ and 24-h dietary recall^16^. A recent GWAS study of dietary habits based on the UK Biobank identified genetic associations between hundreds of dietary habits, providing the potential to study the causal relationship between dietary habits and disease risk^17^. Dietary habits have been widely used to assess causal relationships with diseases or traits, such as migraines^18^, osteoporosis^19^, and cerebral cortex structure^20^.

With the advent of genome-wide association studies and improvements in widely applicable tools, the use of GWAS data for correlation analysis between multiple traits is becoming more common. Linkage disequilibrium score regression (LDSC) is a widely used method to identify genetic correlations among complex traits and to distinguish inflated test statistics from confounding biases and polygenicity in GWAS data^21^. Using the aggregate data from GWAS, LDSC provides a simple and reliable method to screen thousands of traits simultaneously and determine their real genetic correlations^22^. However, LDSC can only analyze genetic correlations between traits. To understand the confounding factors in observational studies and determine causality, Mendelian randomization (MR) can be used with genetic variation as an instrumental variable (IV) to assess whether the observed associations between risk factors and outcomes are consistent with causal effects^23^. MR has been used to identify reliable risk factors for various diseases^24^. The combination of LDSC and MR analyses has been widely used to explore the associations between complex diseases and their risk factors^25^.

In this study, we used LDSC to detect the genetic correlations between dietary habits and AAM. MR analysis assessed the causal relationship between the 143 dietary habits selected and AAM. Our results help to elucidate the potential genetic relationship between dietary habits and AAM.

## Materials and methods

### GWAS summary data

The genetic instruments for dietary habits were acquired from a public GWAS dataset^17^, which included 455,146 individuals from the UK Biobank. All individuals were 40–69 years old and lived in the UK between 2006 and 2010. Dietary habits were assessed using the UK Food Frequency Questionnaire (FFQ)^26^. The information collected included the number of tablespoons of cooked vegetables eaten per day (field 1289), overall oily fish intake (field 1329), and data on foods that were never eaten (from the options dairy, eggs, sugar, and wheat) (field 6144). The GWAS data were processed as follows: Heritability measures were obtained using BOLT-lmm software (v.2.3.2)^27^. Additional covariates in the BOLT-lmm analysis for both heritability and GWAS included the results from genotyping arrays and the first 10 genetic principal components (PCs) derived from a subset of unrelated European individuals using FlashPCA252, followed by projection of related individuals onto the PC space. Principal component analysis (PCA) generated 85 PC-DPs that captured the correlated structures between single food intake information and represented independent components of real-world eating habits^17^. A total of 814 independent loci (defined as > 500 kb apart) were identified, which exceeded the genome-wide significance (*p* < 5.0 × 10^−8^). A full description of the study design, sample characteristics, statistical analysis, and quality control can be obtained from the study results^17^.

GWAS summary data relating to AAM was obtained from the ReproGen Consortium to avoid sample overlap. This dataset^28^ included 182,416 females of European descent from across 58 studies. Individuals who reported their age at menarche as < 9 or > 17 years were excluded from the analysis. Single nucleotide polymorphisms (SNPs) were excluded from the individual study datasets if they were poorly imputed or rare (MAF < 1%). We obtained 3915 SNPs associated with AAM (*p* < 5 × 10^−8^). A full description of the study design, sample characteristics, statistical analysis, and quality control can be obtained from the study results^28^.

### Genetic correlation analysis

The LD score regression (LDSC, v1.0.1, https://github.com/bulik/ldsc) software^21^ evaluated the genetic correlations between 143 dietary habits and AAM. LDSC is a useful approach for estimating the components of heritability and the genetic correlation and has been widely used to analyze complex diseases^21^. After strict Bonferroni correction, *p* < 0.000350 (0.05/143) was considered a significant association. The basic principle of the LDSC method is to use the test statistic of the expected value of the observed χ^2^ SNP under the original hypothesis of no association. SNPs that mark more neighbors—and thus have higher LD scores—are more likely to mark one or more causal loci that influence the phenotype^5^. We restricted our analysis to Hapmap3 SNPs using pre-calculated European LD scores from the 1000 Genomes Project Phase 3 provided by LDSC.

### Selection of instrumental variables (IVs)

The IVs used in MR analysis should meet three conditions: (1) they are correlated with exposure, (2) they are not associated with confounding factors, and (3) they are not related to outcome directly but are related through exposure^29^. The IVs used in this study met the above conditions and are listed in Supplementary Table S2. All variables met the genome-wide significance threshold of *p* < 5 × 10^−8^. The parameters kb = 10,000 and r^2^ = 0.01 were used to remove the linkage disequilibrium between each variable. F-statistics were computed to estimate whether a weak instrument bias was observed and to improve the power of the selected instrumental variables. The F-statistics for all IVs were above the threshold of 10^30^.

### Two-sample MR analysis

A two-sample MR analysis was used to evaluate the causal relationship between dietary habits and AAM. The SNPs used as IVs were within a distance of 10,000 kb and r^2^ > 0.001. A two-sample MR package (version 0.5.6) was used to analyze MR^31^. Five models were used in the MR analysis: (1) the inverse-variance-weighted (IVW) model, (2) the weighted median estimator, (3) the MR-Egger regression method, (4) the simple mode, and (5) the weighted mode. The IVW model was used as the primary method to evaluate the causal effect of dietary habits on AAM. The significance level was taken as *p* < 0.05. Significantly associated dietary habit SNPs were further assessed using statistical analyses, including Cochran’s Q test, a pleiotropy test, and a leave-one-out sensitivity test. However, if the pleiotropy test suggested the presence of pleiotropy (*p* < 0.05), MR Pleiotropy RESidual Sum and Outlier (MR-PRESSO) was used to filter potential outliers and assist in correcting them^32^. Finally, the leave-one-out sensitivity analysis was performed to evaluate whether a single SNP provided significant results.

### Institutional review board statement

Ethical approval was not applicable to our study as publicly available data were used for all analyses.

### Informed consent statement

Informed consent statement was not applicable to our study as publicly available data were used for all analyses.Informed consent was obtained from all subjects involved in the original study.

## Results

### Genetic correlations between 143 dietary habits and AAM

**Figure 1:**
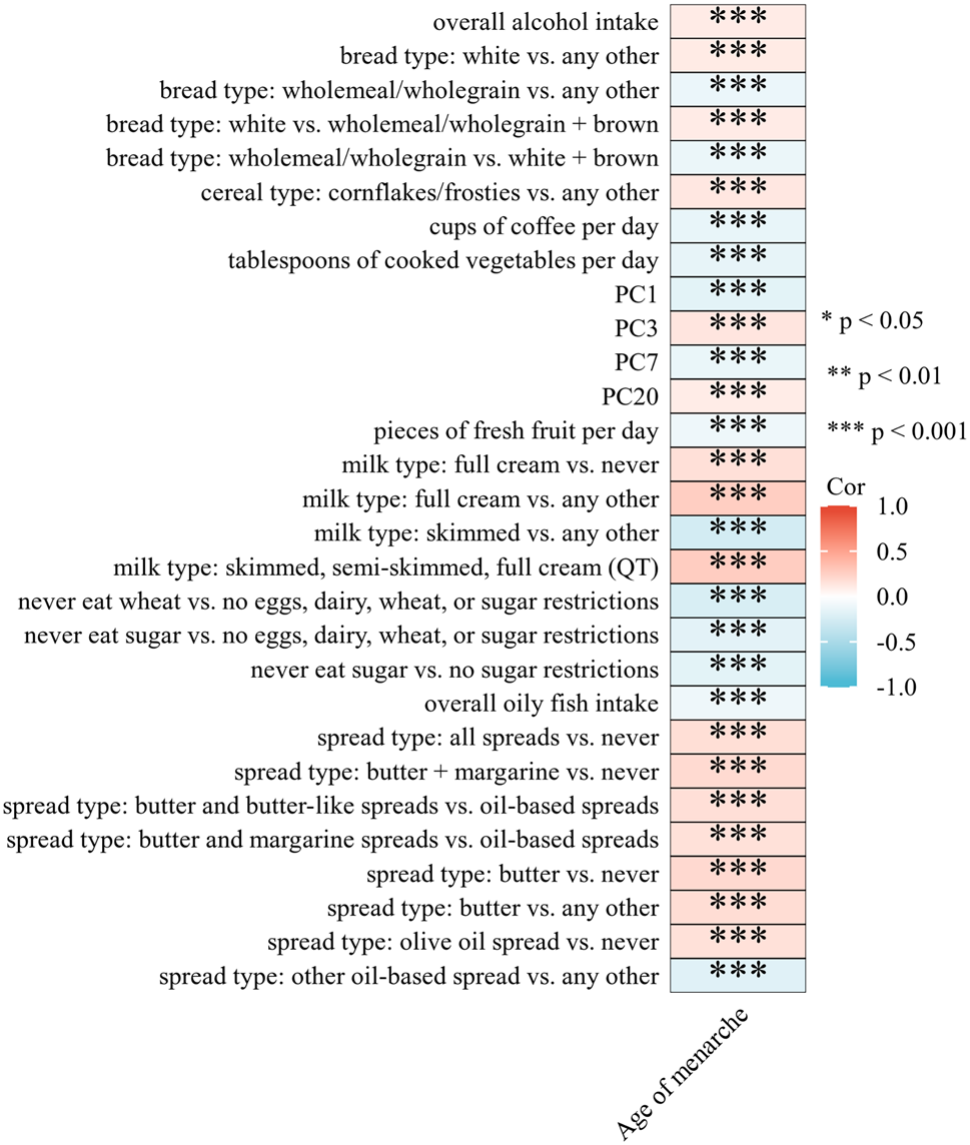
Heatmap of significant correlation (*P*_*ldsc*_ < 3.50 × 10^−4^) of LDSC analysis.

The LDSC analysis identified 29 candidate dietary habits significantly associated with AAM (*P*_*ldsc*_ < 3.50 × 10^−4^, Fig. 1). These included milk types (skimmed, semi-skimmed, and full cream; coefficient = 0.2704; *P*_*ldsc*_ = 1.13 × 10^−14^) and PC1 (coefficient =  − 0.1699; *P*_*ldsc*_ = 1.00 × 10^−10^). PC1 is primarily defined by the type of bread consumed (whole grain/whole meal vs. white bread, two correlated FI-QTs contributing 15.4–15.8%). A further 41 dietary habits showed a suggestive association with AAM (0.05 < *P*_*ldsc*_ < 3.50 × 10^−4^), such as the frequency of adding salt to food (coefficient = 0.0862, *P*_*ldsc*_ = 6.00 × 10^−4^), PC24 (coefficient = 0.1093, *P*_*ldsc*_ = 5.00 × 10^−4^), and overall poultry intake (coefficient =  − 0.0945, *P*_*ldsc*_ = 3.80 × 10^−3^). All genetic correlations between the 143 dietary habits and AAM are summarized in Supplementary Table S1.

### Causal relationships between 143 dietary habits and AAM

**Figure 2:**
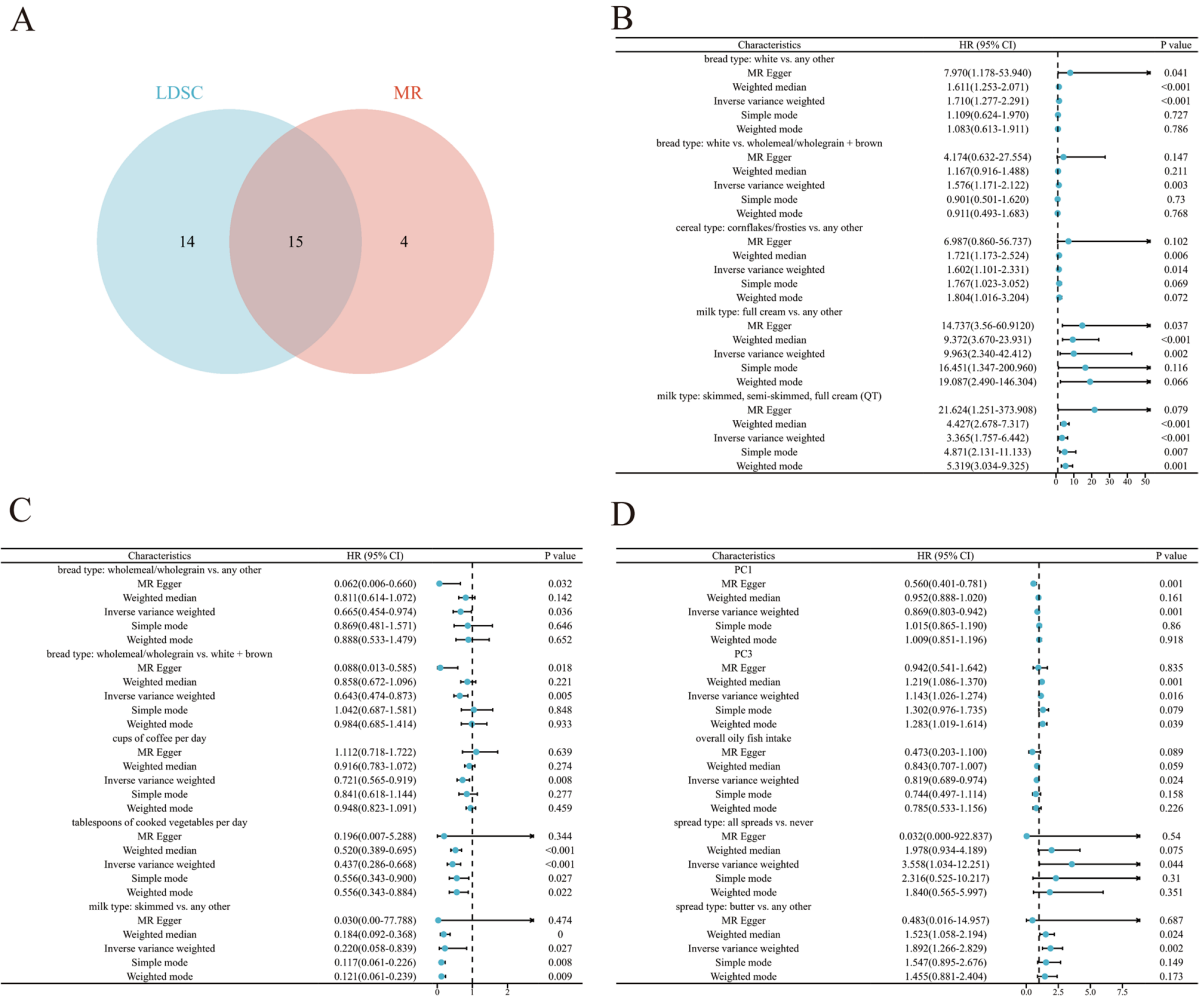
Results of MR analysis. (**A**) Veen plot of LDEC and MR. (**B**–**D**) Forest plots of MR results. *MR* Mendelian randomization, *LDSC* Linkage disequilibrium score regression, *CI* confidence interval.

The MR study identified causal relationships between 19 dietary habits and AAM. In addition, 15 dietary habits showed genetic correlations with AAM and therefore were deemed to have causal relationships with AAM. These included bread type (white vs. any other; OR 1.71, 95% CI 1.28–2.29; *P*_*mr*_ = 3.20 × 10^−4^), tablespoons of cooked vegetables per day (OR 0.437, 95% CI 0.29–0.67, *P*_*mr*_ = 1.30 × 10^−4^), milk type (skimmed, semi-skimmed, and full cream; OR 3.37, 95% CI 1.76–6.44, *P*_*mr*_ = 2.50 × 10^−4^), cups of coffee per day (OR 0.72, 95% CI 0.57–0.92, *P*_*mr*_ = 8.31 × 10^−3^), and PC3 (OR 1.14, 95% CI 1.03–1.27, *P*_*mr*_ = 0.01) (Fig. 2, Supplementary Tables S2–S3). PC3 is primarily defined by the spread type (butter vs. any other, one correlated FI-QT contributing over 10%)^17^.

### Sensitivity analysis

**Figure 3:**
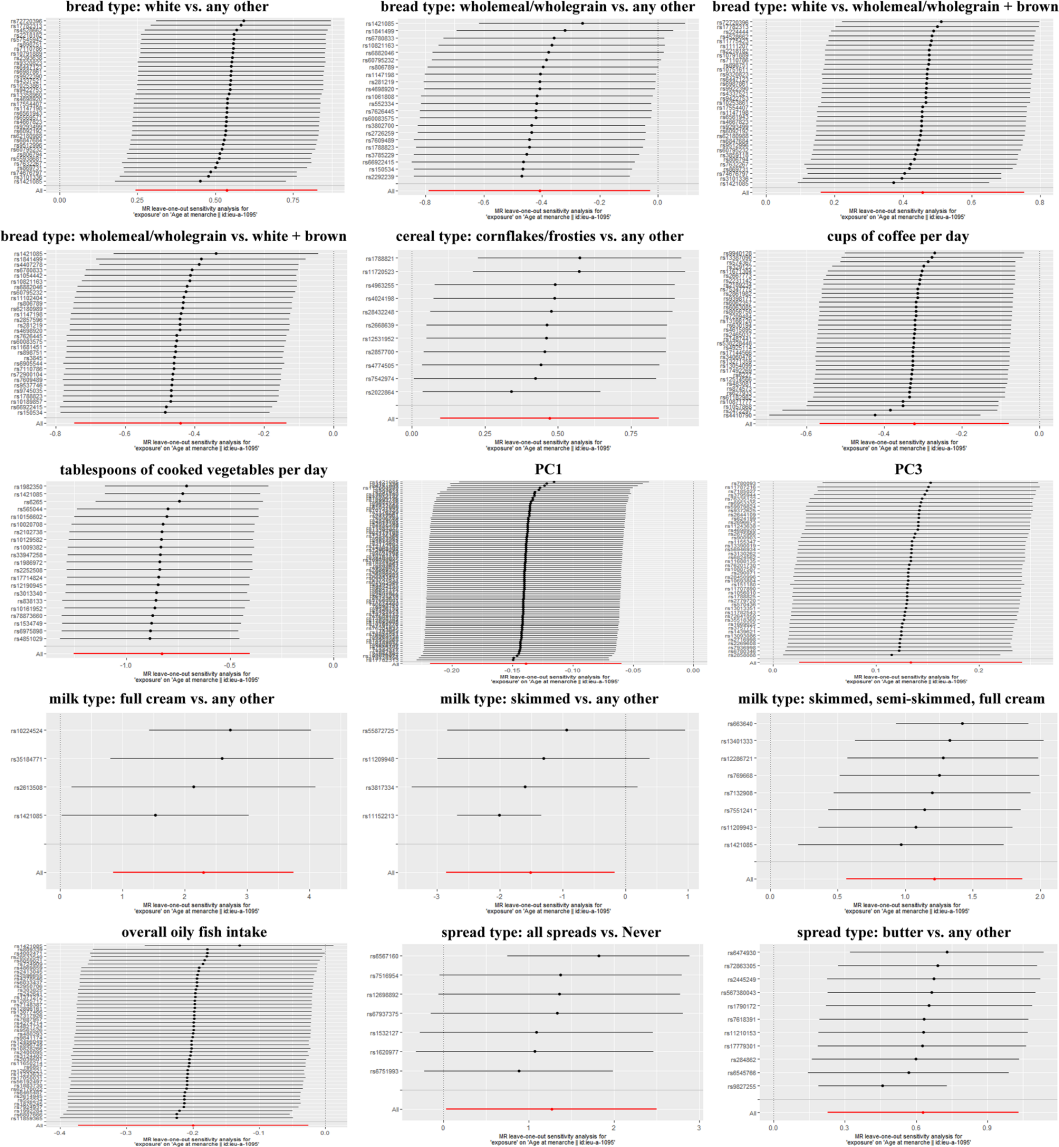
Leave-one-out sensitivity test.

All dietary habits identified as significantly associated with AAM were further analyzed using Cochran’s Q test (Supplementary Table S4), the pleiotropy test (Supplementary Table S6), and the leave-one-out sensitivity analysis. The results of these tests were used to inform the MR method. In the absence of heterogeneity and pleiotropy, estimated IVW results were preferentially used, thus this method was used most frequently in this study. When there was only heterogeneity, but no pleiotropy, a weighted median or random-effect IVW was used (Supplementary Table S5). Although a few results were heterogeneous, the direction of the effect obtained from these other methods was concordant with the IVW results. However, the MR-Egger method was used if the pleiotropy test suggested that the result was multi-efficacious. The leave-one-out sensitivity test results suggested the result should be considered reliable (Fig. 3, Supplementary Information 2).

## Discussion

In this study, we performed LDSC and MR analyses to investigate the relationship between dietary habits and AAM. Using LDSC analysis, we identified 29 candidate dietary habits significantly associated with AAM. We further evaluated the causal relationship between dietary habits and AAM using MR analysis. After sensitivity analyses, we identified eight dietary habits that showed significant causal relationships with AAM: bread type (whole meal, whole grain, white, brown), cereal type (cornflakes, frosties), milk type (skimmed, semi-skimmed, full cream), spread type (butter, margarine, olive oil), overall oily fish intake, tablespoons of vegetables per day, and PC1 and PC3, which primarily represent bread type and spread type, respectively.

Diet plays a key role in human health, and a recent study showed that diet-associated risk is among the top five risks of attributable deaths worldwide^33^. Dietary habits affect physical health in childhood^34^ and adulthood, especially concerning chronic diseases^35^. A pooled analysis of 2181 population-based studies^36^ showed that lifelong health advantages and risks are affected by heterogeneous nutritional quality. Diet influences the age of puberty onset and further affects height gain during adolescence and late adolescence. Another prospective study of 3983 children showed that puberty started later in children with a high diet quality^37^. A prospective cohort study of 215 girls showed that girls with a low intake of white meat (poultry and fish), fruits, and vegetables had an earlier AAM^38^. In addition to dietary patterns, single dietary components can also affect AAM. A cohort study among Chinese children suggested that the higher the childhood soy intake, the later the puberty^39^. According to Cheng TS. et al., a higher dietary fat intake has been associated with earlier puberty^40^. Although many cohort studies have observed the effects of different diets on AAM, the underlying mechanisms have not yet been elucidated. The effect of diet on AAM is influenced by many confounding factors, which are often difficult to control^41^. Therefore, the current research on the relationship between diet and AAM is mainly observational, and the results are often controversial. MR Analysis has a unique advantage in excluding confounding factors^42^. Although Cheng TS. et al. showed a causal effect of higher dihomo-γ-linolenic acid concentrations on earlier AAM using MR analysis^43^. There have been no studies on the association between dietary habits in general and AAM. We used the most comprehensive dietary habit GWAS summary dataset to evaluate the relationship between dietary habits and AAM from a genetic perspective.

The effect of estrogenic endocrine disruptors (EEDs) on puberty has long been understood^44^. Numerous observational studies have shown that girls chronically exposed to EEDs are more likely to experience early puberty^45–47^, regardless of the incidence of obesity^48^. The similar structure of EEDs to estrogen allows them to bind and activate estrogen receptors, thus exerting effects similar to estrogen^49^. Kisspeptin regulates puberty and fertility in humans^50^ by stimulating GnRH neuronal activity and regulating ovulation by driving luteinizing hormone surges^51^. Exposure to EEDs affects the expression of kisspeptin and GnRH and influences the pulsatile release of GnRH, which subsequently alters gonadotropin levels^52^. Animal studies have shown that EEDs alter hypothalamic Kiss1 mRNA expression levels and kisspeptin fiber density while altering gonadotropin secretion and/or gonadotropin-releasing hormone neuronal activation^53^. Coffee is one of the most widely consumed beverages worldwide^54^ and contains various ingredients, including caffeine, carbohydrates, lipids, and proteins, and has a few estrogenic activities^55^. In addition to caffeine, other components of coffee, such as aromatic acids, esters, and sterols, have estrogenic activity^56^. Our results suggest that increased daily coffee intake leads to earlier AAM, which may be attributed to the estrogenic activity of coffee. Isoflavones as EEDs have the potential to modulate estrogen metabolism^57^. Isoflavones are structurally similar to estrogens and can compete with endogenous estrogen for the estrogen receptor^58^. Whole wheat bread contains high concentrations of isoflavones (average 450 mcg/100 g)^59^ and is the primary source of isoflavones in the European diet^60^. Thus, our results suggest that the intake of whole grain bread may result in earlier AAM compared to that associated with white bread. Similarly, increased daily vegetable intake was associated with earlier AAM. The consumption of phytoestrogen-containing vegetables, such as soy and soy products, may promote puberty onset by performing estrogen-like biological functions^61,62^. The intake of vegetables, especially raw vegetables, may increase the risk of pesticide exposure^63,64^, and the effects on puberty timing as a type of EED have been extensively studied^65,66^.

Insulin-like growth factors (IGFs), including IGF-1 and IGF-2, are important peptides that regulate essential cellular activities^67^. IGF-1 levels are associated with age and play a key role in growth^68^. A few longitudinal studies have shown that higher IGF-1 levels are related to earlier puberty onset^69^ and higher breast cancer risk^70^. Oily fish were positively associated with circulating IGF-I concentrations^71^. Our results suggest that an increased intake of oily fish may lead to earlier AAM. In addition, several prospective cohort studies have shown that milk intake before puberty may accelerate AAM^72,73^. Other studies have shown no effect of milk intake on AAM^74,75^. Although milk contains IGF-1, studies have shown that humans do not absorb biologically significant levels of intact IGF-I from their food^76^. Different types of milk may contain different levels of sugar, fats, proteins, and other trace elements. A study based on the Growth and Obesity Cohort showed that consumption of sugar-sweetened milk beverages led to earlier mammary gland development compared to that in low-fat dairy products, namely low-fat milk and yogurt^77^. Another study showed that milk and butter consumption at the ages 3–5 was inversely related to breast development at age 10.8^78^. Our results suggest that butter intake was negatively associated with AAM, while the intake of full-cream milk did not lead to earlier AAM compared to skimmed or semi-skimmed milk. The controversy over these results is mainly due to the complex composition of milk beverages^79^. Therefore, stricter control of milk composition in the study cohort would be needed to produce more rigorous results. Cultural factors may play a confounding role; for example, girls with earlier AAM may avoid milk if they believe it is associated with acne, while girls with later AAM may consume more milk and grow further in height. These factors may lead to uncertainty in the causal associations determined by such studies.

Despite the lack of longitudinal studies, this work is suggestive of new directions for understanding the genetic mechanisms between diet habits and AAM, since we are the first to combine LDSC and MR analysis to investigate the causal association between dietary habits and AAM. However, our study has some limitations. Although eating habits may change in adulthood compared to childhood, eating habits formed during childhood have been shown to have a lasting effect on adult eating habits^80^. We used data of eating habits in adulthood to reflect the exposure to certain products in childhood, due to the lack of large-scale heritability studies of childhood eating habits. First, dietary recall errors and individual differences in dose estimates are inevitable in an FFQ. Such reporting errors may lead to underestimating the true relationship between dietary habits and AAM. Second, people with AAM < 9 years or > 17 years were excluded. Therefore, the conclusions of this study are limited to people with AAM within the typical range and do not apply to people with abnormal pubertal initiation (precocious puberty or delayed pubertal development). Further, only European populations were included in this study, and as such, the conclusions should be interpreted with caution concerning other ethnic groups. Finally, the results of LDSC and MR only suggest possible genetic correlations and causal associations from a genetic perspective. Further experimentation is needed to confirm any underlying biological mechanisms indicated by these genetic associations. It is worth noting that the interpretation of genetic factors in dietary traits can be complex. Especially when AAM is also influenced by environmental factors. Therefore, more GWAS data on children and lifestyle questionnaires are expected to be generated. This will facilitate the study of genetic associations of lifestyle in children.

## Conclusions

Based on GWAS summary data for dietary habits and AAM, we identified 29 candidate dietary habits that showed genetic associations with AAM. MR evaluations revealed that 19 dietary habits were causally associated with AAM, including bread type (whole meal, whole grain, white, brown), cereal type (cornflakes, frosties), milk type (skimmed, semi-skimmed, full cream), spread type (butter, margarine, olive oil), overall oily fish intake, and tablespoons of vegetables per day. These results provide a potential novel understanding of the genetic mechanisms underlying the relationship between diet habits and AAM.

### Supplementary Information

Supplementary Information 1.

Supplementary Tables.

## Data Availability

The datasets analyzed during the current study are available from the UK biobank (http://geneatlas.roslin.ed.ac.uk/) (fields: 20002), ReproGen Consortium (https://www.reprogen.org/data_download.html).
