# Supplementary material for: Genetic correlation and Mendelian randomization analyses support causal relationships between dietary habits and age at menarche
Source: Sci Rep. 2024 Apr 10;14:8425. doi: 10.1038/s41598-024-58999-4 (PMC11006932; doi:10.1038/s41598-024-58999-4)

Leave-one-out Plots for the MR Analyses

1. among current drinkers, drinks usually with meals: yes + it varies vs. No

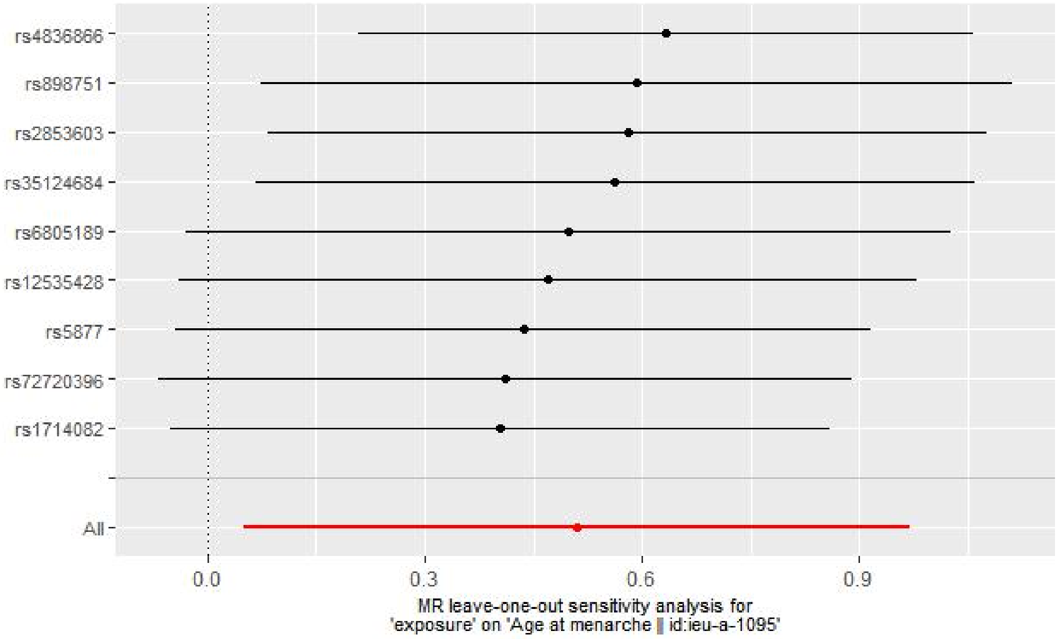

2. bread type: white vs. any other

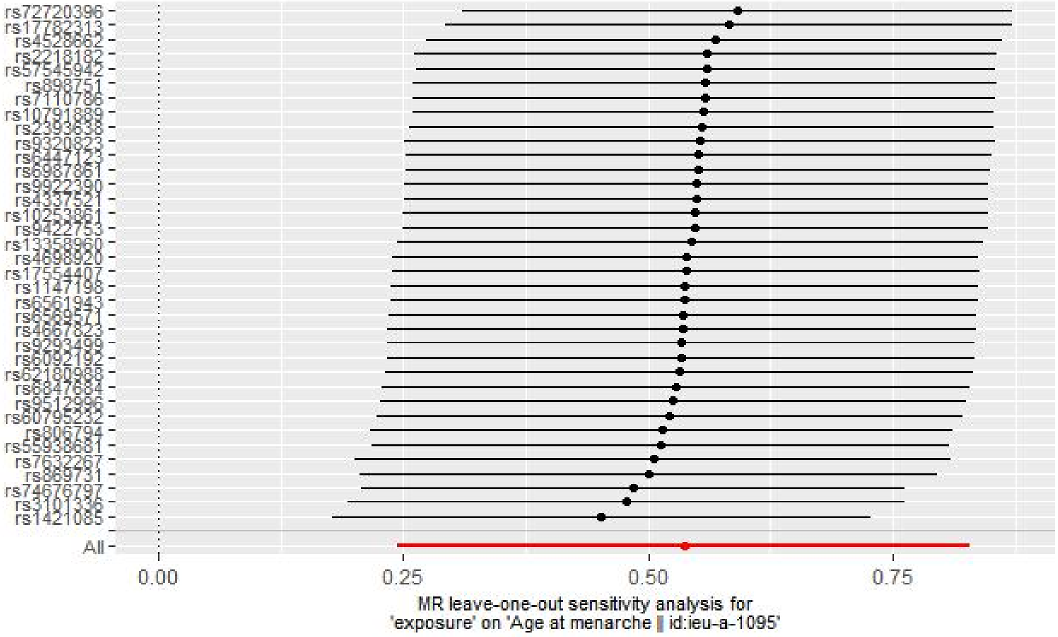

3. bread type: wholemeal/wholegrain vs. any other

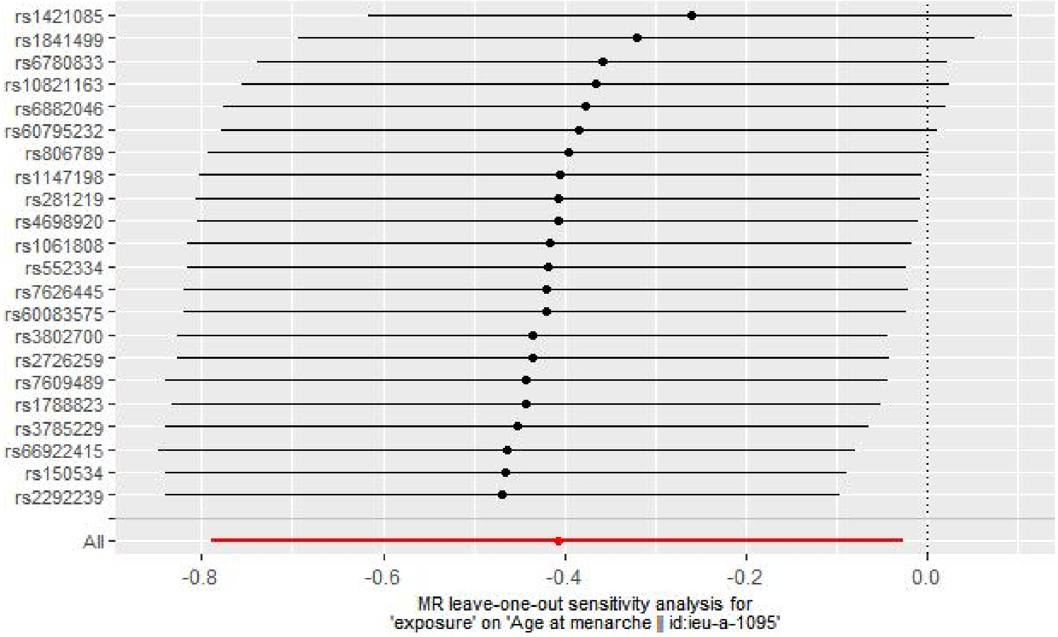

4. bread type: white vs. wholemeal/wholegrain + brown

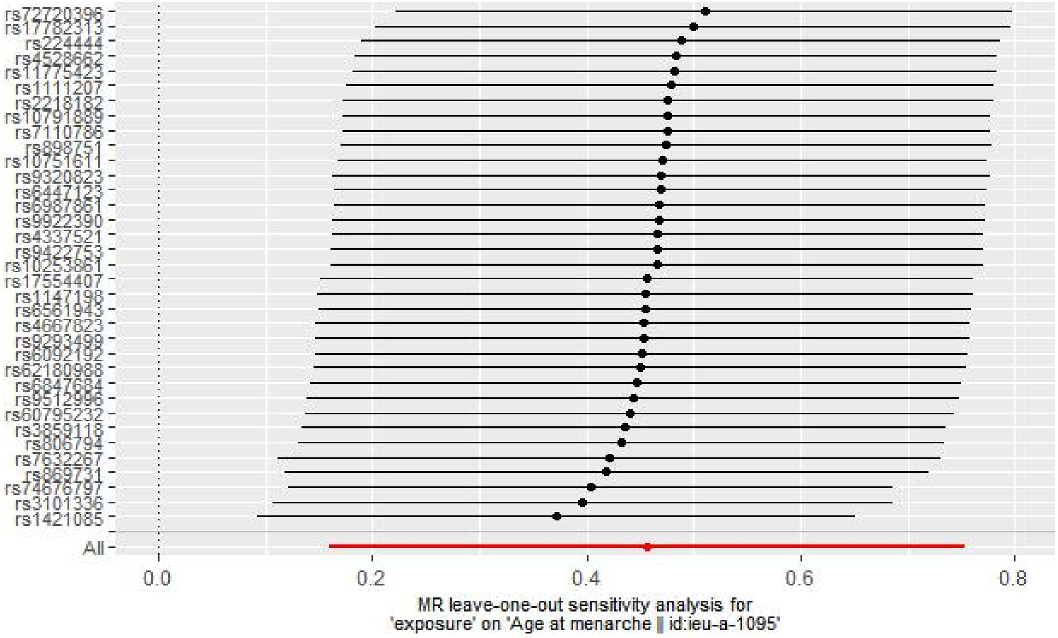

5. bread type: wholemeal/wholegrain vs. white + brown

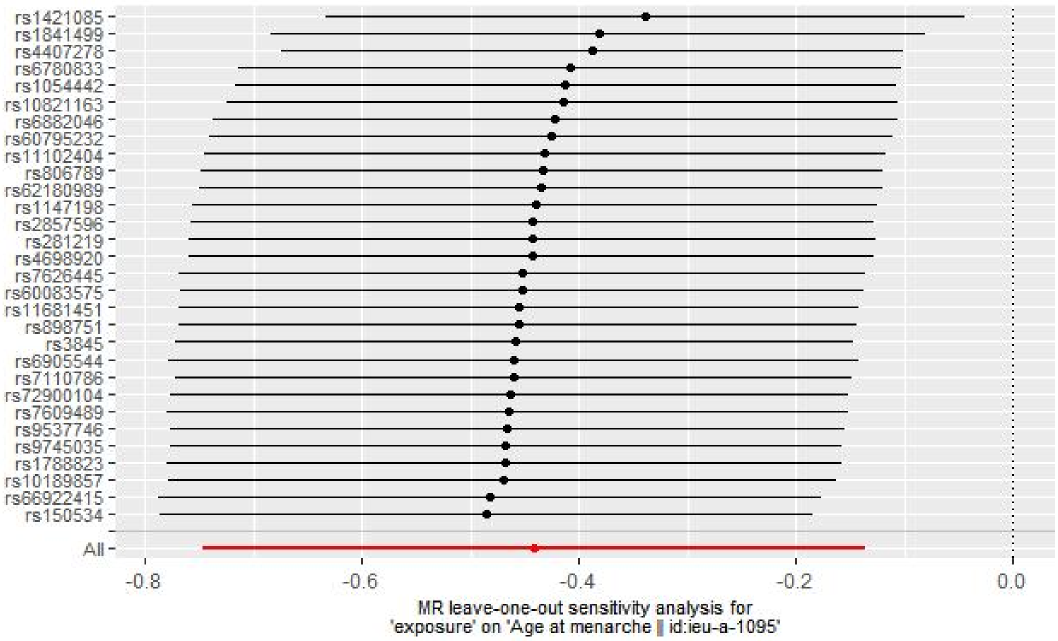

6. cereal type: cornflakes/frosties vs. any other

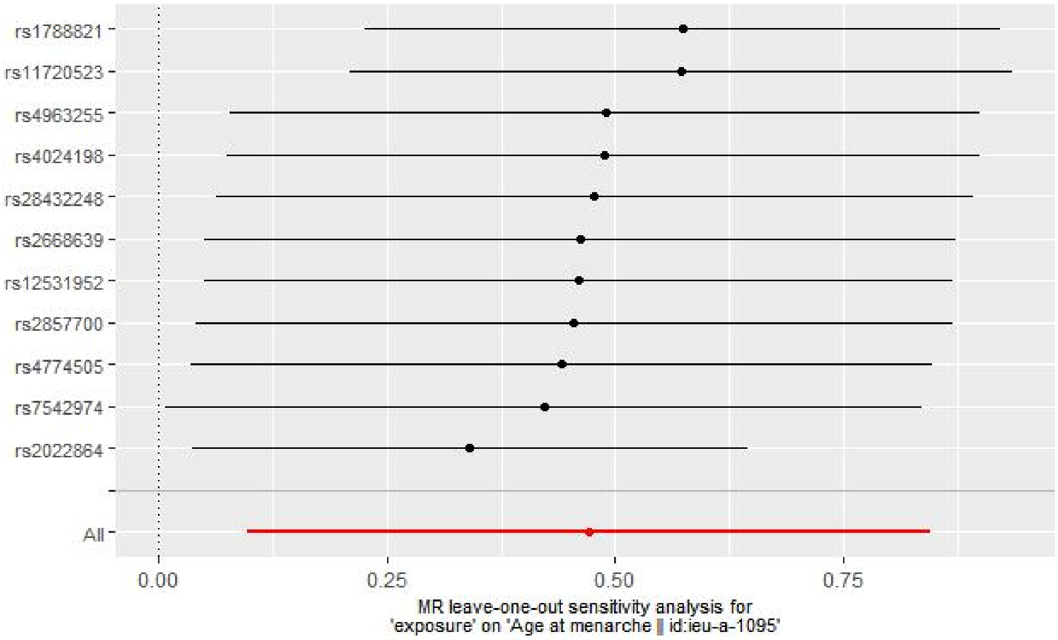

7. cups of coffee per day

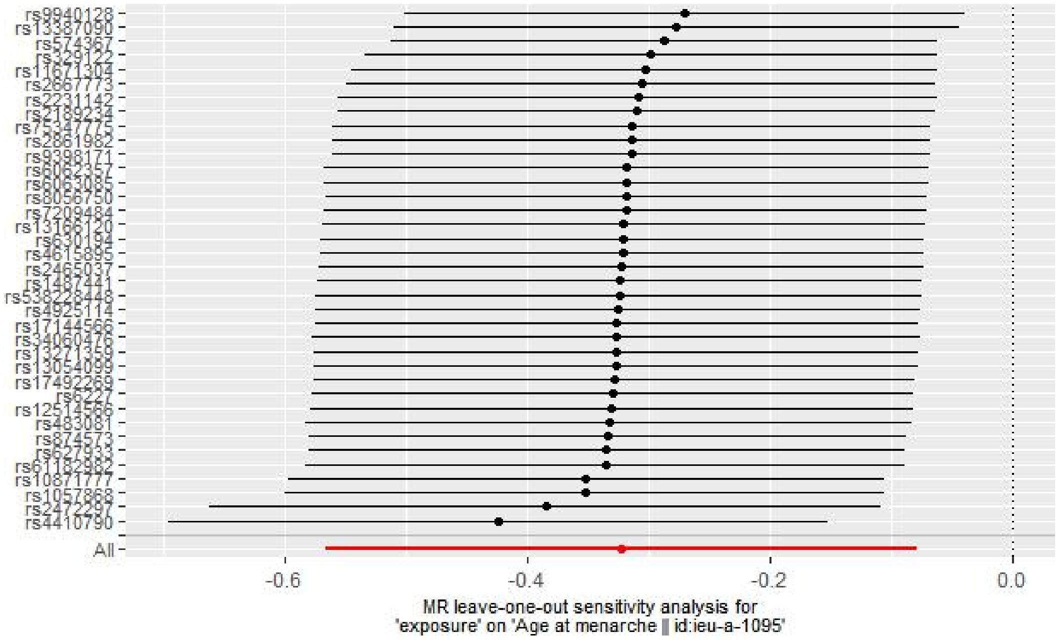

8. tablespoons of cooked vegetables per day

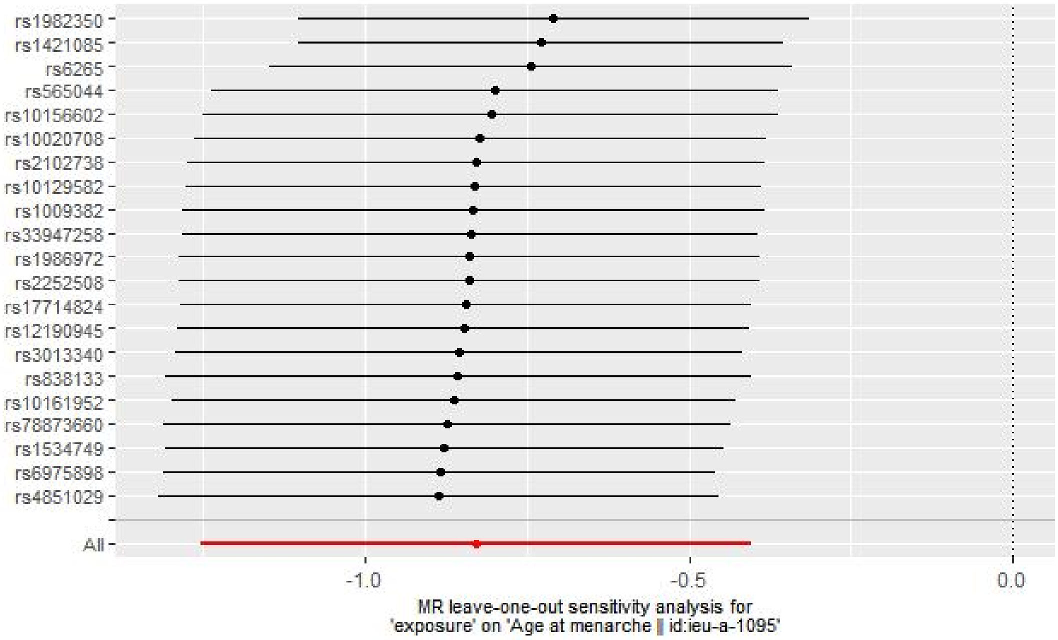

9. PC1

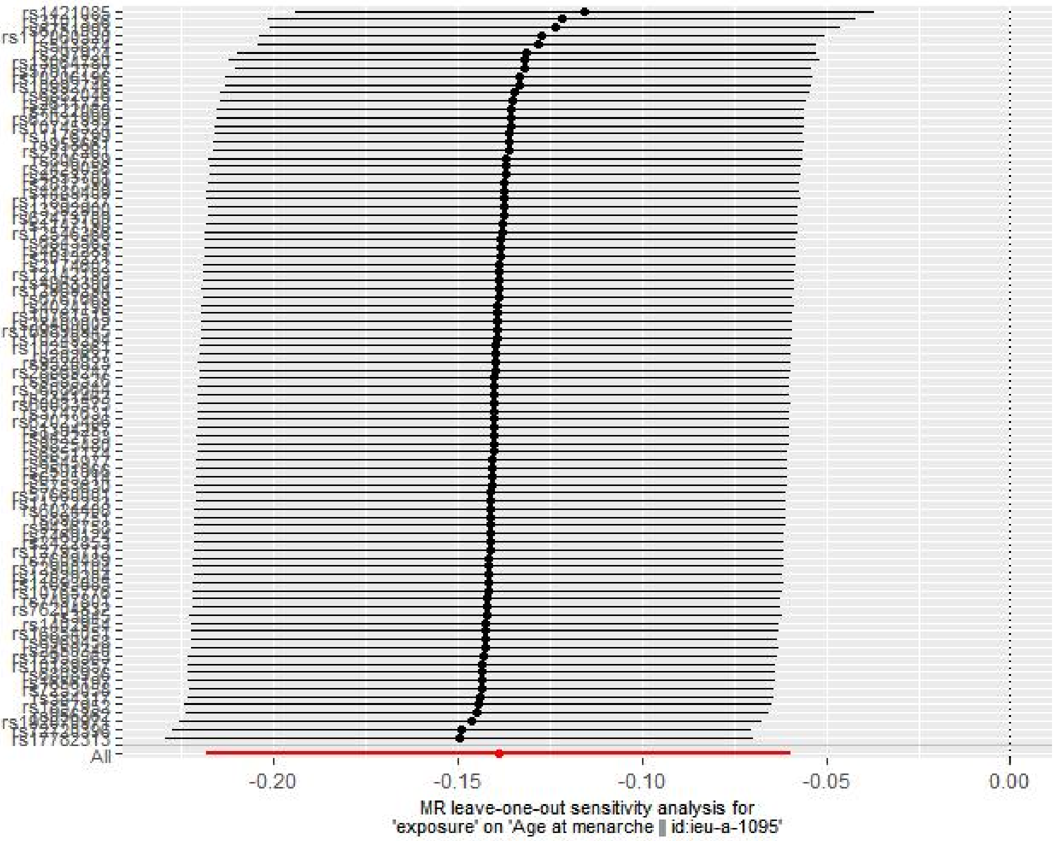
rs1421085
rs17782313
All
-0.20
-0.15
-0.10
-0.05
0.00
MR leave-one-out sensitivity analysis for
'exposure' on 'Age at menarche || id:ieu-a-1095'

10. PC3

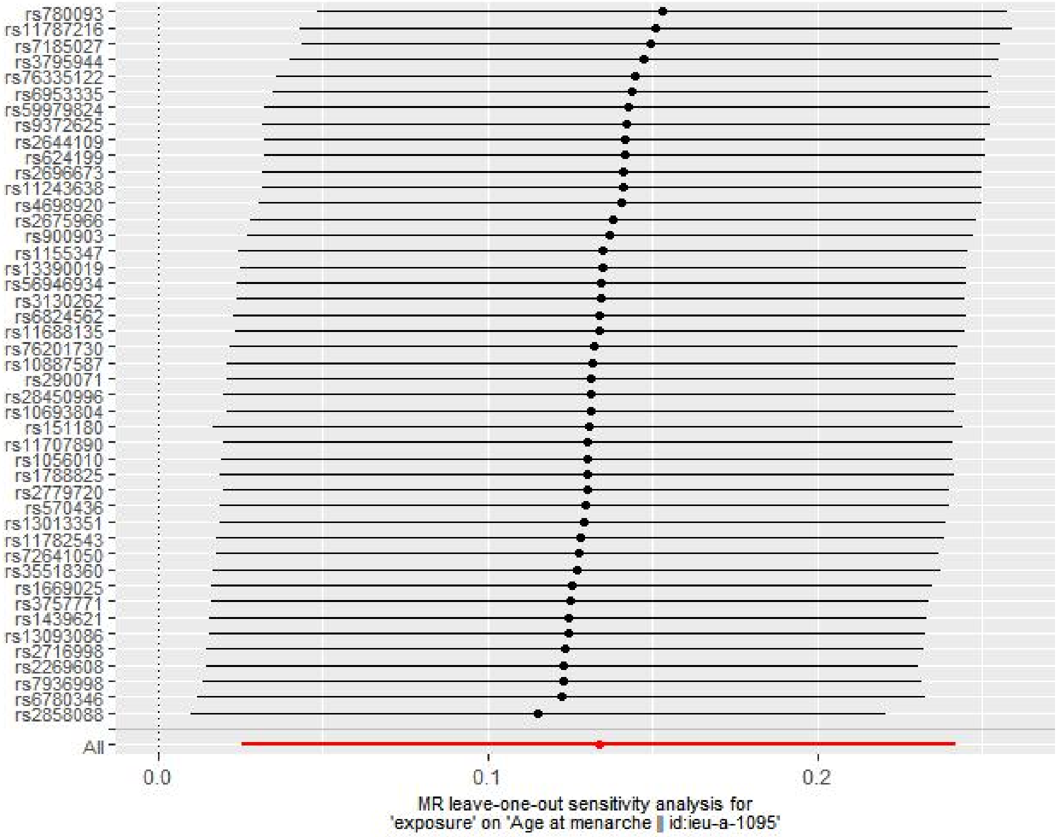
rs780093
rs11787216
rs7185027
rs3795944
rs76335122
rs6953335
rs59979824
rs9372625
rs2644109
rs624199
rs2696673
rs11243638
rs4698920
rs2675966
rs900903
rs1155347
rs13390019
rs56946934
rs3130262
rs6824562
rs11688135
rs76201730
rs10887587
rs290071
rs28450996
rs10693804
rs151180
rs11707890
rs1056010
rs1788825
rs2779720
rs570436
rs13013351
rs11782543
rs72641050
rs35518360
rs1669025
rs3757771
rs1439621
rs13093086
rs2716998
rs2269608
rs7936998
rs6780346
rs2858088
All
0.0
0.1
0.2
MR leave-one-out sensitivity analysis for
'exposure' on 'Age at menarche || id:ieu-a-1095'

11. PC19

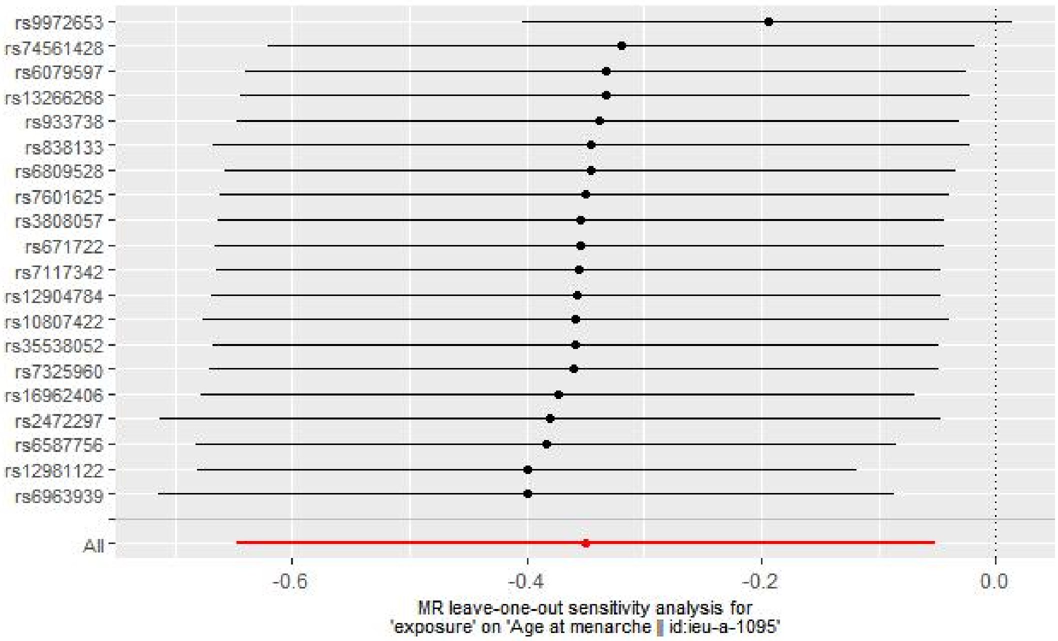

12. milk type: full cream vs. any other

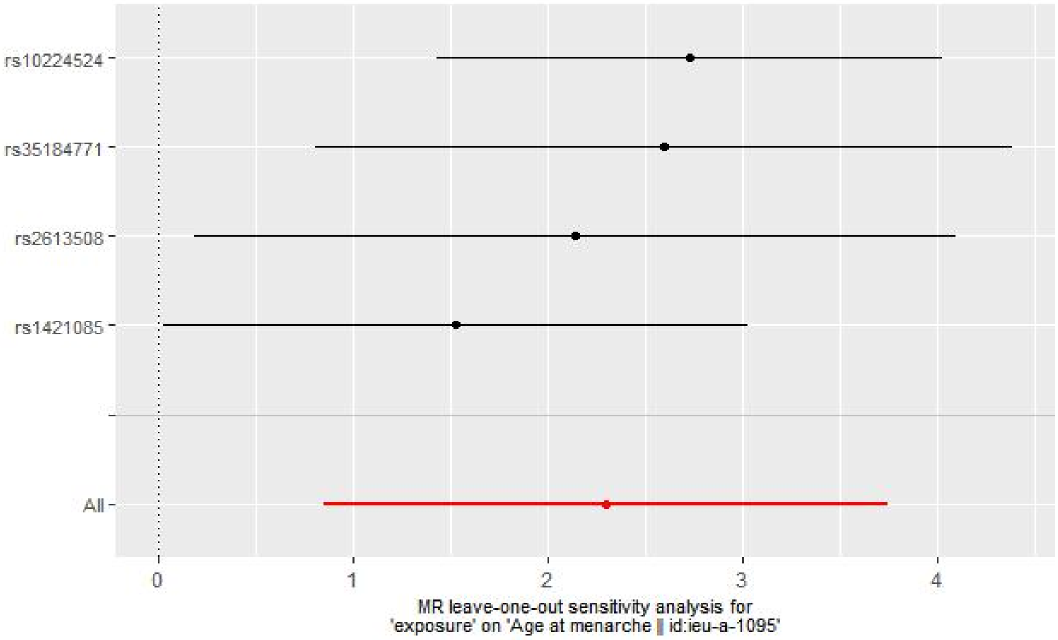

13. milk type: skimmed vs. any other

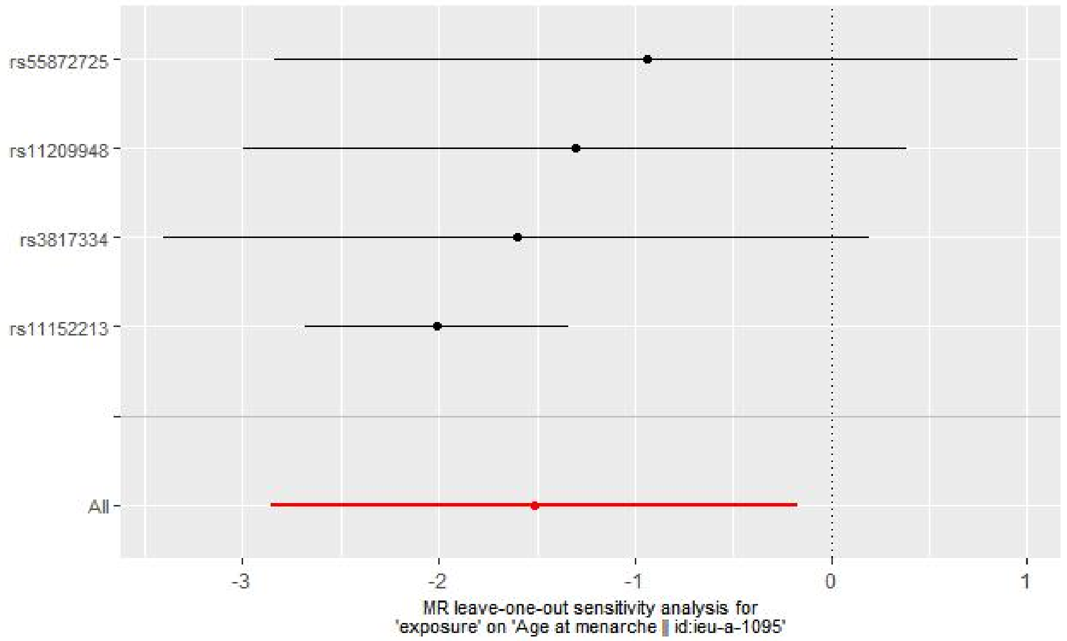

14. milk type: skimmed, semi-skimmed, full cream (QT)

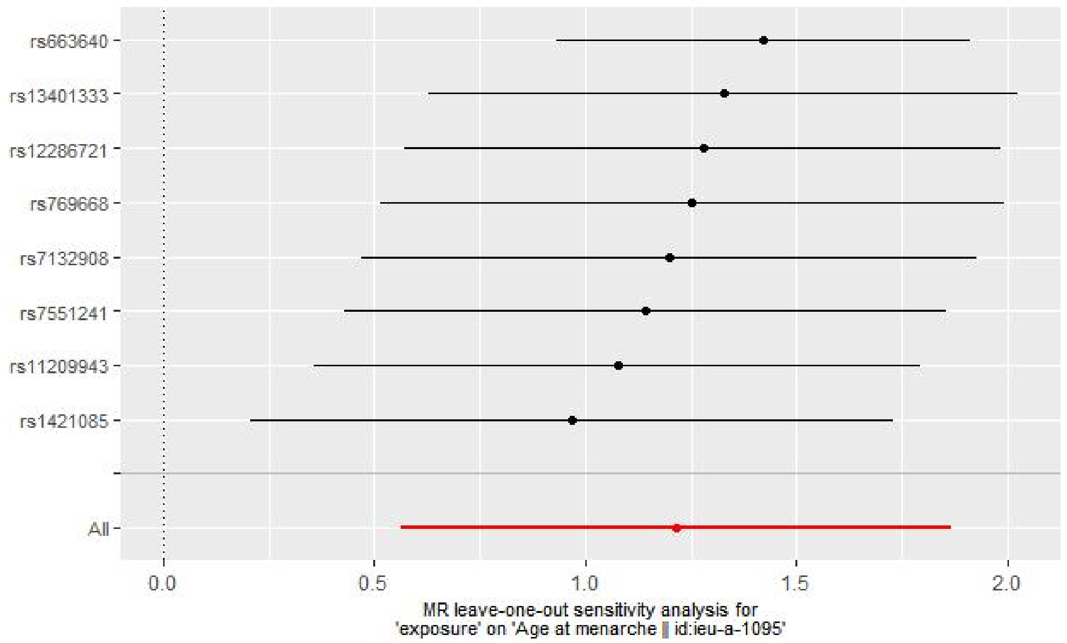

15. overall oily fish intake

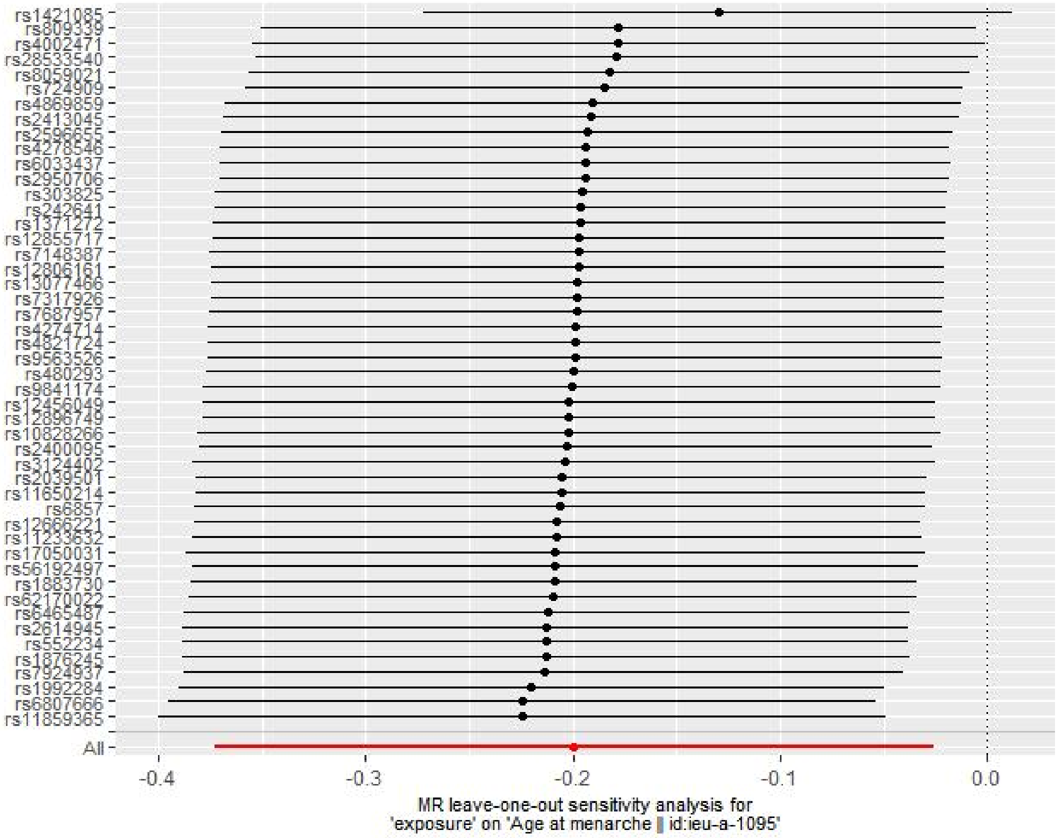

16. tablespoons of raw vegetables per day

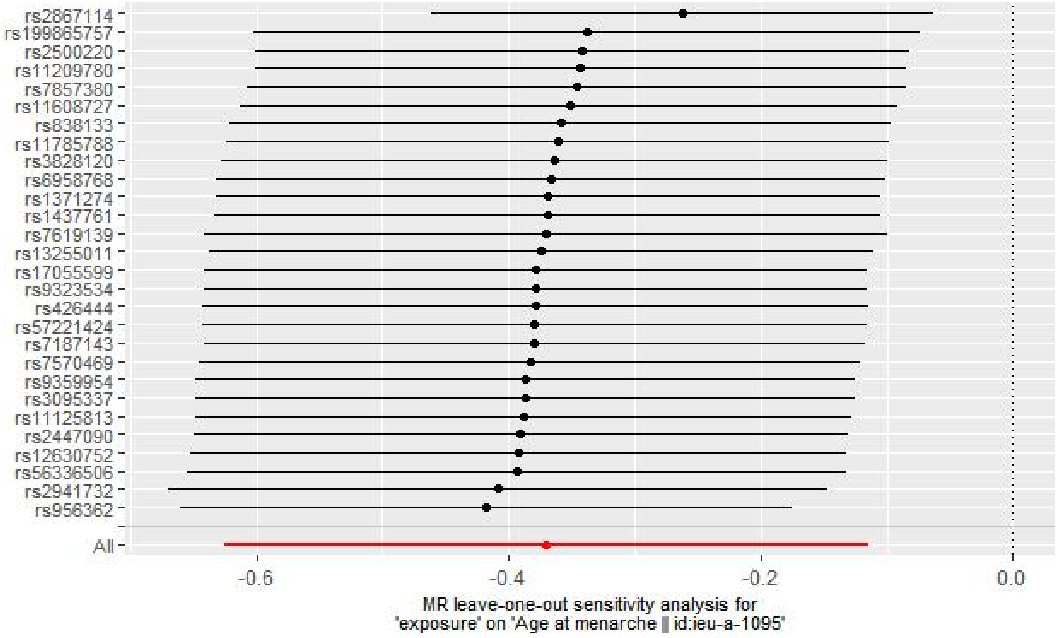

17. spread type: all spreads vs. Never

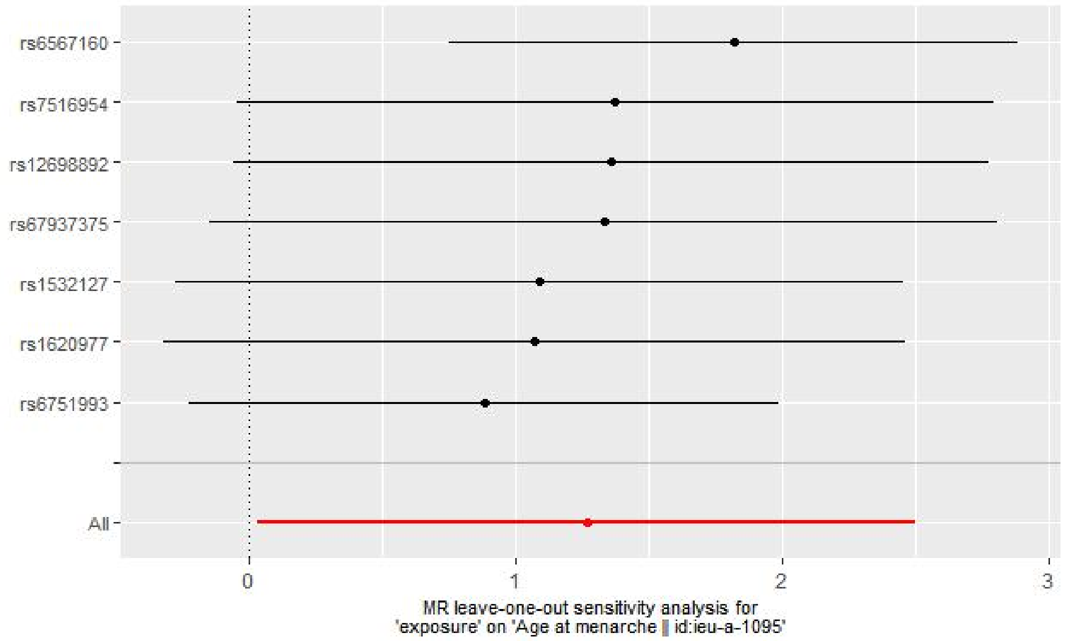

18. spread type: butter vs. any other

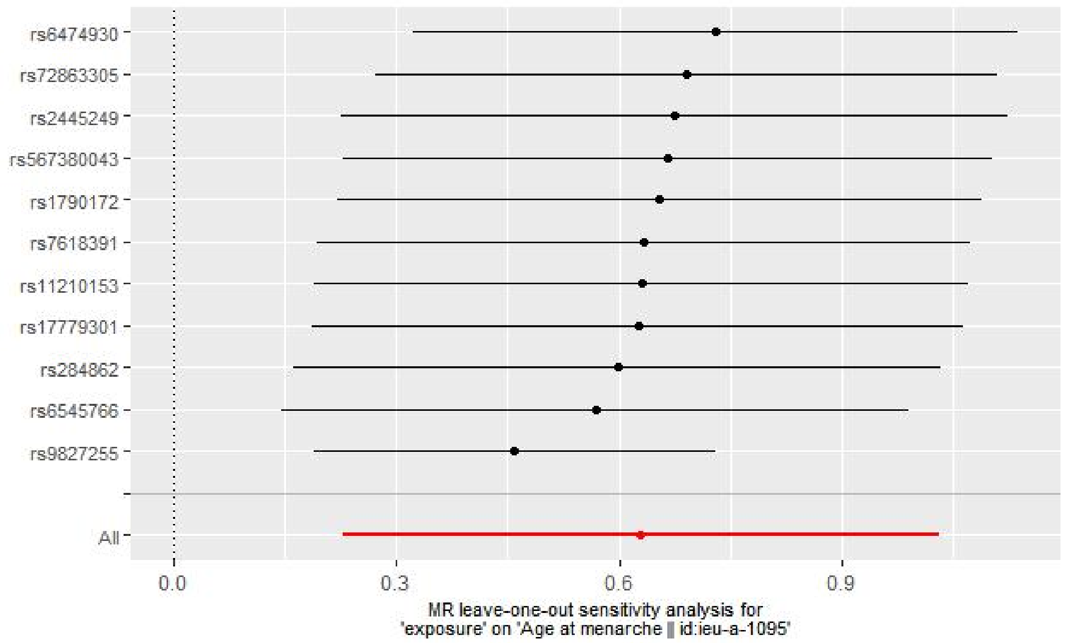

19. glasses of water per day

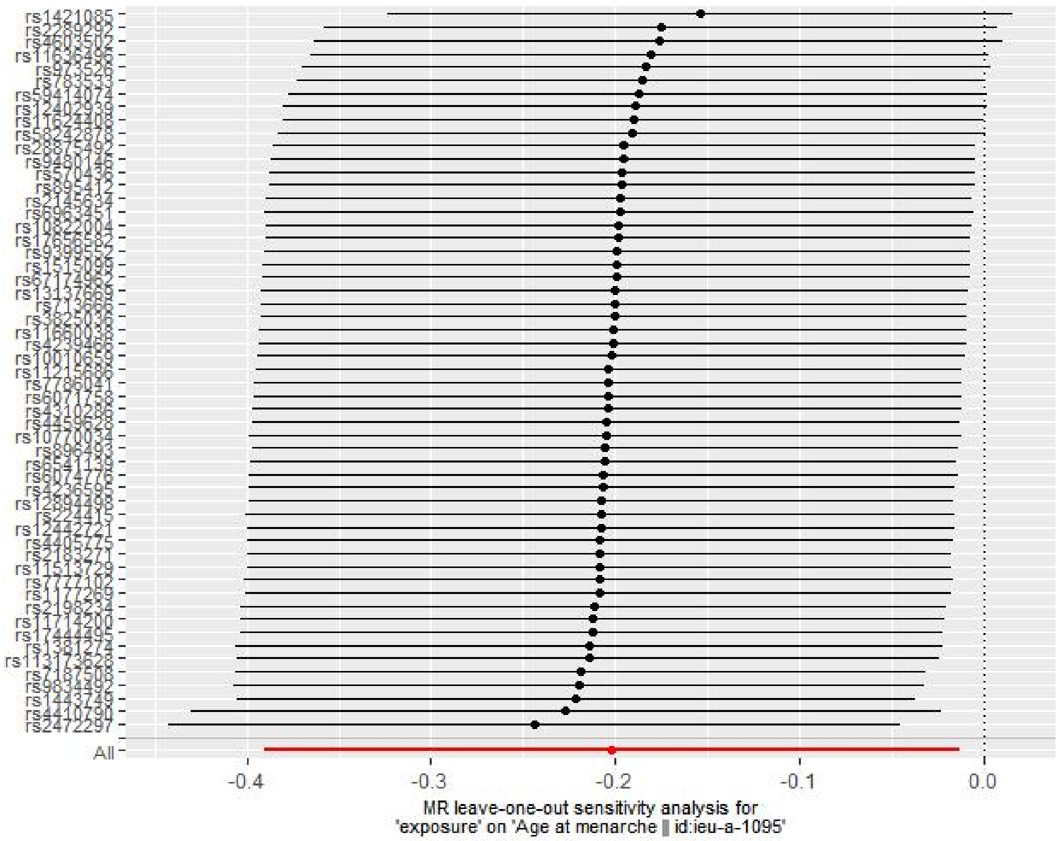

Supplement: Supplementary file 1 — Supplementary Information 1. [file 41598_2024_58999_MOESM1_ESM.pdf]
